# Supplementary material for: Exploring molecular variation and combining ability of local and exotic bread wheat genotypes under well-watered and drought conditions
Source: PeerJ. 2025 Feb 27;13:e18994. doi: 10.7717/peerj.18994 (PMC11874937; doi:10.7717/peerj.18994)
Supplement: Supplemental Information 1 [file peerj-13-18994-s001.docx]

**Table S1.** Code, name, and pedigree of eight wheat genotypes utilized in this study.

| **Parent** | **Name** | **Pedigree** |
| --- | --- | --- |
| **P1** | Gemmeiza12 | OTUS./3/.SARA/.THB//VEECMSS97Y00227S-5y-010M 010Y-010M 2Y-1M-0Y OGM |
| **P2** | Sids12 | BUC.//7C/.ALD/5/.MAYA74/.ON//.1160-147/3/ BB-/GLL/4/ CHAT"S" /6/MAYA /VUL // CMH74 A. 630/4*SX |
| **P3** | Line-117 | CIMMYT/C. 2008/29ESWYT/OCC. 549/Plot141/ Rep1/Block 9/Entry 142 |
| **P4** | Line-144 | CIMMYT/C. 2008/29ESWYT/OCC. 549/Plot136/Rep1/Block 8/Entry 136 |
| **P5** | Line-123 | SOKOLL/WBLL1 |
| **P6** | Line-125 | CHEN/.AE.SQ.//2*-WEAVER./3/BAV92./4/JARU./5/OLI2./SALMEJA /6/CROC-1/AE-SQUARROSA(205)-//-BORL-95/3/PRL./SARA//TSI./VEE#5/4/FRET2 |
| **P7** | Gemmeiza7 | CMH74630./5X//SERI82./3/.AGENTCGM4611 2GM.3GM.1GM.0GM |
| **P8** | Line-121 | CHEN./AE-SQ.//2*OPATA./3/FINSI |

**Table S2.** Physical and chemical soil properties of experimental sites across the two growing seasons.

| **Properties** | **Season 1** | **Season2** |
| --- | --- | --- |
| Soil texture | Clay | Clay |
| Sand (%) | 14.70 | 13.30 |
| Silt (%) | 32.40 | 32.20 |
| Clay (%) | 52.90 | 54.50 |
| pH | 8.52 | 8.32 |
| EC (dS/m) | 3.74 | 3.43 |
| Organic matter (%) | 1.95 | 1.86 |
| Available Nitrogen (N) (mg kg^-1^) | 38.58 | 36.85 |
| Available phosphorus (P) (mg kg^-1^) | 11.46 | 10.76 |
| Available potassium (K) (mg kg^-1^) | 262.63 | 248.80 |

Table S3. Heterosis relative to better parent for all studied traits under well-watered (NOR) and water-deficit (WAD) conditions.

| **Cross** | **Days**  **to heading** | | **Plant height**  **(cm)** | | **Spike length**  **(cm)** | | **No. of spikelets/**  **spike** | |
| --- | --- | --- | --- | --- | --- | --- | --- | --- |
|  | NOR | WAD | NOR | WAD | NOR | WAD | NOR | WAD |
| P 1×P 2 | 4.98** | 6.67** | 6.44 | 13.22* | -8.57* | -8.20 | -4.92 | -11.98** |
| P 1×P 3 | -3.17* | -1.08 | -2.86 | -0.46 | -15.32** | -13.90** | -12.02** | -6.94* |
| P 1×P 4 | 0.00 | 0.36 | -5.49 | 1.71 | -7.28 | -5.53 | -6.42 | -7.14* |
| P 1×P 5 | -0.71 | 1.11 | -3.77 | -11.21* | -12.69** | -12.76** | -11.20** | -13.78** |
| P 1×P 6 | 2.17 | -1.10 | -7.26 | -6.57 | -11.65** | -8.88* | -12.57** | -13.49** |
| P 1×P 7 | 2.16 | 2.91 | -11.32** | -8.71 | -3.17 | -4.78 | -22.13** | -23.75** |
| P 1×P8 | 0.71 | 0.72 | -6.69 | -10.88* | -3.14 | -1.82 | -7.79* | -4.99 |
| P 2×P 3 | 6.51** | 7.45** | 6.66 | 11.06* | -12.35** | -21.06** | -15.74** | -28.11** |
| P 2×P 4 | 5.75** | 2.75 | -1.89 | -1.69 | 0.84 | -0.68 | -5.84 | -15.94** |
| P 2×P 5 | 6.51** | 5.88** | 6.08 | 9.06 | -12.69** | -13.19** | -16.20** | -19.34** |
| P 2×P 6 | 6.90** | 7.45** | 6.93 | 7.15 | -7.10 | -4.63 | -5.03 | -16.51** |
| P 2×P7 | 9.20** | 7.45** | 2.90 | 9.56 | -1.39 | 0.58 | -1.89 | -20.75** |
| P 2×P8 | 8.43** | 7.06** | 3.98 | 12.55* | -1.13 | -2.78 | -10.15** | -17.36** |
| P 3×P 4 | -1.40 | -0.72 | 11.55* | 12.07* | -2.91 | -15.00** | -0.93 | -4.37 |
| P 3×P 5 | 2.49 | 3.69* | 10.29* | 15.15** | 6.38 | 12.38* | -5.78 | -8.41* |
| P 3×P6 | 3.62* | 0.37 | 4.75 | 12.30* | 0.92 | -1.10 | -7.37 | -13.11** |
| P 3×P 7 | -0.36 | 0.36 | 5.73 | 12.10* | -0.40 | -0.71 | -11.76** | -6.63 |
| P 3×P8 | 2.14 | -0.36 | 16.97** | 17.90** | 4.96 | -0.76 | 1.26 | 5.18 |
| P 4×P5 | 1.07 | 2.95 | -6.29 | -3.27 | 7.16 | -1.59 | 2.17 | 2.19 |
| P 4×P6 | 1.45 | -3.30 | 4.66 | 3.75 | 4.77 | -5.07 | -0.15 | -0.94 |
| P 4×P7 | -3.24* | -5.45** | -0.27 | 3.37 | 8.44* | 0.68 | -3.52 | 0.90 |
| P 4×P8 | 1.07 | 2.17 | 5.49 | 6.89 | 9.96* | -1.44 | 4.04 | 6.25 |
| P 5×P6 | 2.17 | 1.11 | 6.01 | 6.57 | 4.07 | 11.53* | 2.89 | 6.30 |
| P 5×P7 | -1.08 | 0.74 | 2.22 | 7.49 | 2.44 | 2.43 | -4.80 | -3.92 |
| P 5×P8 | 1.43 | 2.58 | -8.57* | -6.51 | 7.84 | 1.02 | -1.42 | 1.63 |
| P 6×P7 | 0.00 | 0.73 | 11.94** | 7.78 | 2.28 | 0.00 | -1.59 | -1.20 |
| P 6×P8 | -2.17 | -2.20 | 8.75* | 2.16 | -5.48 | -11.68* | -5.27 | 0.99 |
| P 7×P8 | 0.00 | 0.00 | 1.89 | 3.01 | 4.08 | -1.90 | -6.84 | -7.23* |

Table S4. Cont.

| **Cross** | **No. of spikes /plant** | | **No. of kernels / spike** | | **1000-kernel weight**  **(g)** | | **Grain yield/plant**  **(g)** | |
| --- | --- | --- | --- | --- | --- | --- | --- | --- |
|  | NOR | WAD | NOR | WAD | NOR | WAD | NOR | WAD |
| P 1×P 2 | -17.20 | -1.11 | -5.24 | -8.77* | 11.47* | -3.33 | -3.79 | 9.23 |
| P 1×P 3 | -21.83 | -9.47 | -11.52** | -7.02 | -0.66 | -3.92 | -19.81* | 5.68 |
| P 1×P 4 | -15.85 | 3.01 | -8.90** | -7.02 | 2.27 | -6.46 | -12.14 | -13.68 |
| P 1×P 5 | -7.16 | -3.72 | -4.19 | -20.47** | 6.26 | -10.64 | 28.26** | 36.86** |
| P 1×P 6 | -31.10* | -22.11 | -14.14** | -5.26 | -11.75* | -20.82** | -27.39** | -23.77 |
| P 1×P7 | -18.70 | -38.72* | -19.90** | -24.56** | -7.41 | -14.16* | -28.26** | -22.29 |
| P 1×P8 | -0.27 | -16.27 | -6.98* | -4.68 | 6.65 | -12.84* | -11.54 | -32.75** |
| P 2×P 3 | -29.31* | -39.21** | -17.49** | -20.37** | 1.97 | 7.56 | -24.43** | -45.11** |
| P 2×P 4 | -13.13 | -31.49 | -3.28 | -12.35** | 7.52 | -8.36 | 9.33 | -29.80** |
| P 2×P 5 | -16.43 | -47.27** | -6.01 | -12.35** | 8.60 | -11.99* | 24.50* | -0.68 |
| P 2×P 6 | -15.10 | -26.84 | -16.39** | -8.02* | 10.15 | -6.05 | -4.87 | -8.73 |
| P 2×P7 | -21.59 | -42.03* | -7.53* | -24.24** | -1.85 | -9.25 | -1.91 | -4.91 |
| P 2×P8 | -19.61 | -51.97** | -7.65* | -9.88* | -2.44 | -14.32** | -0.79 | -30.26** |
| P 3×P 4 | -3.62 | 7.89 | -3.49 | -10.00** | 5.32 | -19.39** | -13.99 | -19.10 |
| P 3×P 5 | -6.44 | -13.15 | -5.78 | -8.39* | -5.05 | -8.32 | 7.51 | 25.33* |
| P 3×P 6 | -2.70 | 18.42 | -6.36 | -13.55** | 9.18 | 1.80 | -5.54 | 6.46 |
| P 3×P7 | -16.29 | -17.54 | -10.75** | -5.45 | -5.56 | -4.63 | -2.60 | -22.07 |
| P 3×P8 | 14.79 | 13.66 | 16.76** | 16.13** | 4.27 | 5.87 | 22.56* | 31.63** |
| P 4×P 5 | -16.26 | -15.63 | 4.77 | 5.88 | 5.51 | 6.03 | 13.34 | 21.92 |
| P 4×P6 | 36.96** | -12.82 | 1.68 | 1.76 | -2.57 | 4.03 | -0.96 | 15.12 |
| P 4×P 7 | -2.36 | 2.52 | -5.38 | -7.65* | 1.67 | 12.46* | -21.24* | -2.23 |
| P 4×P8 | -18.14 | -14.09 | 3.40 | 0.59 | 1.71 | -10.58 | 3.48 | 11.10 |
| P 5×P6 | -22.16 | -33.67* | 0.00 | -2.58 | 1.93 | -1.25 | 8.43 | -2.84 |
| P 5×P7 | -0.36 | -15.51 | -4.39 | -3.03 | -8.15 | -5.47 | 17.14 | 36.32* |
| P 5×P8 | -2.83 | -12.78 | -1.74 | 1.29 | -10.30* | -8.32 | 1.57 | 10.92 |
| P 6×P7 | 0.43 | -8.95 | -0.54 | -0.61 | -2.16 | 1.49 | 13.21 | 35.68** |
| P 6×P8 | -18.44 | -33.91* | 4.62 | 0.00 | 8.29 | -18.77** | -11.24 | -10.34 |
| P 7×P8 | 12.08 | -13.54 | -6.45* | -6.67 | 0.79 | 2.97 | 1.12 | -32.84** |
